# CircRNA_0079586 and circRNA_RanGAP1 are involved in the pathogenesis of intracranial aneurysms rupture by regulating the expression of MPO

**DOI:** 10.1038/s41598-021-99062-w

**Published:** 2021-10-05

**Authors:** Zhuang Zhang, Rubo Sui, Lili Ge, Dongjian Xia

**Affiliations:** 1 Department of Neurology, The First Affiliated Hospital, Jinzhou Medical University, Jinzhou, China; 2 Department of Ultrasound, The First Affiliated Hospital, Jinzhou Medical University, Jinzhou, China; 3 Department of Neurosurgery, The First Affiliated Hospital, Jinzhou Medical University, Renmin Street, No.2, Fifth Duan, Jinzhou, China

**Keywords:** Cell biology, Molecular biology

## Abstract

Several circRNAs have been reported to be dysregulated in human endothelial cells through sponging miRNAs. Previous reports demonstrated that MPO not only contributed to the formation and rupture of cerebral aneurysm but was also correlated with the degenerative remodeling predisposition to saccular intracranial aneurysm wall rupture, although its underlying mechanisms remain to be explored. Microarray screening was performed to compare the differential expression of circRNAs in the endothelial cells collected from UIAs and RIAs patients. Luciferase assays were used to explore the regulatory relationship between circRNAs and miRNAs, and between miRNAs and their target genes. Microarray screening analysis found a batch of up-regulated circRNAs in the endothelial cells harvested from RIAs patients, including circRNA-0079586 and circRNA-RanGAP1. Luciferase assays revealed the suppressive role of miR-183-5p/miR-877-3p in the expression of circRNA-0079586/circRNA-RanGAP1/MPO. And the expression of circRNA-0079586 and circRNA-RanGAP1 was respectively suppressed by the overexpression of miR-183-5p and miR-877-3p. And both the transfection of miR-183-5p and miR-877-3p mimics suppressed the relative expression level of MPO mRNA. The expression of circRNA-0079586, circRNA-RanGAP1 and MPO was significantly activated in the endothelial cells collected from RIAs patients when compared with UIAs patients, whereas the expression of miR-183-5p and miR-877-3p was remarkably suppressed in the endothelial cells collected from RIAs patients when compared with UIAs patients. We further altered the expression of circRNA-0079586 and circRNA-RanGAP1 using siRNA and overexpression in HUVECS, and the expression of circRNA-0079586 and circRNA-RanGAP1 was significantly and negatively correlated with the expression of miR-183-5p and miR-877-3p, but positively correlated with the expression of MPO under different conditions. In this study, we established two MPO-modulating signaling pathways of circRNA_0079586/miR-183-5p/MPO and circRNA_RanGAP1/miR-877-3p/MPO. These two signaling pathways are involved in the pathogenesis of intracranial aneurysms rupture.

## Introduction

Intracranial aneurysm (IA) rupture is an extensively examined topic. Also, unruptured IA is reported in 5% of adults globally^1–3^. Although the rate of ruptured IA is actually not high, ruptured IA can lead to significant consequences such as death as well as disability^4,5^. On the contrary, current IA treatments are associated with significant risk. Therefore, the accurate evaluation of IA rupture is vital for the clinician to consider surgery risk as well as the danger of natural IA rupture^6^. For many years, methods such as geometry, size, as well as location analyses of aneurysm have been used to study the risk factors of IA rupture^7,8^. While large size aneurysms have been thought to be associated with a higher risk of rupture, latest researches have actually shown that a lot of ruptured aneurysms are small in dimensions^8^.

Shear stress has been considered a significant factor for platelet activation, while other factors like levels of collagen, ADP, as well as fibrinogen also play a role^9–11^. Under high shear stress, platelets can also be more easily activated for a brief period of time^12,13^. In terms of how wall shear stress (WSS) causes aneurysm rupture, it is strongly believed that the absolute value of WSS alone may not be used to reasonably forecast the onset of aneurysm rupture, because it does not involve any type of directional information. Physiological researches have also revealed that the endothelial cells of blood capillaries can respond to the changes in the direction as well as magnitude of WSS, and such responses may cause the onset of aneurysm re-modeling and additional growth and rupture of the aneurysm^7,14,15^.

Circular RNAs (circRNAs) belong to a subfamily of endogenous non-coding RNAs (ncRNAs) and are mainly consisted of exonic transcripts produced via back splicing^16^. Different from linear RNAs, circRNA is a covalently closed continuous loop without 5′–3′ polarity or a polyadenylated tail which commonly originates from protein-coding genes and complete exons^17^. And it was reported that eukaryotic circRNAs are mainly produced during splicing, catalyzed by spliceosomal machinery or by groups I and II ribozymes^18^. Moreover, circRNA is also known as miRNA sponge, which regulates the expression of miRNAs. Recent research has actually shown that circRNAs are richly expressed in cells to serve as a necessary regulator in transcriptional as well as post-transcriptional gene expression^19^. In colon cancer patients, circRNA CDR1as is significantly overexpressed in tumor tissues, while the silencing of CDR1as hinders the development of tumors by targeting miRNA-7^20^.

In a previous report, it was failed to show that MPO is linked to the clinical risk of IA rupture^21^. Therefore, it was proposed that the presence of MPO might indicate a more delicate wall of aneurysm that is prone to IA rupture. The level of MPO can be identified using enhanced MRI or blood samples collected from IA fundus. It was also suggested that MPO might be used as a biomarker to examine the risk of IA rupture^22,23^. Also, the measurement of MPO might improve the assessment of cardiovascular diseases. Moreover, it was suggested that myeloperoxidase (MPO) played a key role in inflammation as well as oxidative stress, and is involved in the onset and development of atherosclerosis. And circRNAs have been reported to take key regulatory roles in pathological biological processes such as inflammation and cell proliferation^24^. Besides, miR-183-5p was demonstrated to suppress inflammatory responses in intracerebral hemorrhage mice^25^, while miR-887-3p was found to lead to dysregulated inflammation and enhance lung injury by increasing the cytokine production in ICU patients with severe sepsis^26^.

Several circRNAs have been reported to be dysregulated in human endothelial cells^27,28^. Specially, circRNA_0079586 was found to accelerate the progression of glioma via interacting with miR-183-5p, while circRNA_RanGAP1 was found to facilitate gastric cancer invasion and metastasis via interacting with miR-877-3p^29,30^. Moreover, previous reports demonstrated that MPO not only contributed to the formation and rupture of cerebral aneurysm, but was also correlated with the degenerative remodeling predisposition to saccular intracranial aneurysm wall rupture^22,31^. In this study, we aimed to study the molecular mechanism underlying the role of MPO and its potential signaling pathways.

## Materials and methods

### Human subjects sample collection

In this study, we recruited 34 IA patients and divided them into two groups based on their status of IA: RIAs (ruptured intracranial aneurysms, N = 18) and UIAs (unruptured intracranial aneurysms, N = 16). Intracranial aneurysm tissue samples were collected after removal surgery from patients in both groups following previous published methods^32,33^. Endothelial cells were isolated from surgically removed intracranial aneurysm. The clinical features including sex, age, hypertension, smoking history, lesion size were collected from the patients in the two groups and compared using Student’s t tests. The Human Research Ethics Committees of the First Affiliated Hospital of Jinzhou Medical University has approved this research. All methods were performed in accordance with the last vision of the Declaration of Helsinki. Written informed consent was obtained from all patients before the study.

### Primary culture of endothelial cells from human carotid artery

EC primary culture (Cell applications, San Diego, CA) was derived from human carotid arteries, and the characteristics of the EC primary culture were confirmed by immunohistochemistry.

### Loading shear stress

To measure the loading shear stress, the primary ECs were cultured on glass slides coated with gelatin overnight and then loaded with a shear stress of 0.05 and 3.0 Pa. After 24 h of culturing under the loaded shear stress, the primary ECs were harvested for other analyses. Additionally, the primary ECs were also subjected to the treatment with a turbulent flow as previously described^27^.

### Genotyping by direct sequencing

Total RNA of the samples was first extracted by using a Trizol reagent (Invitrogen, Carlsbad, CA) according to the directions of the reagent manufacturer. Then, the extracted total RNA was subsequently processed using DNase I (Takara, Dalian, China) according to the directions of the reagent manufacturer to get rid of genomic DNA contaminants. In the next step, equal volumes of total RNA extracted from each sample were subject to deep sequencing, which was carried out by BGI (Shenzhen, China) by making use of an Illumina Solexa platform.

### Microarray analysis

To examine the circRNA expression profile in collected tissue and cell samples, the total RNA, which also contained circRNAs and miRNAs, in each sample was isolated and studied using the Affymetrix GeneChip miRNA 2.0 microarray (Affymetrix, Santa Clam Clara, CA) according to the directions of the microarray manufacturer. Based on the results, circRNAs identified with changed expression were divided into 2 groups, i.e., a group of circRNAs with upregulated expression and a group of circRNAs with downregulated expression. The group of circRNAs with upregulated expression included 9 circRNAs: circRNA_0004543 (2.6-fold increase in expression), circRNA_0079586 (4.1-fold increase in expression), circRNA_0000231 (3.5-fold increase in expression), circRNA_0003204 (2.7-fold increase in expression), circRNA_0454542 (3.3-fold increase in expression), circRNA_0091822 (3.5-fold increase in expression), circRNA_ RanGAP1 (3.9-fold increase in expression), circRNA_0943034 (3.1-fold increase in expression), and circRNA_320434 (1.9-fold increase in expression). The group of circRNAs with downregulated expression included 6 circRNAs: circRNA_0003492 (2.1-fold decrease in expression), circRNA_0011032 (2.5-fold decrease in expression), circRNA_0004264 (3.2-fold decrease in expression), circRNA_0002331 (3.7-fold decrease in expression), circRNA_0004528 (2.8-fold decrease in expression), and circRNA_0000345 (2.6-fold decrease in expression).

### RNA isolation and real-time PCR

RNA isolation and real-time PCR were carried out to study the expression of circRNA_0079586, circRNA_RanGAP1, miR-183-5p, miR-877-3p, and MPO mRNA in each sample. In brief, total RNA in each sample was extracted using a mirVana RNA extraction assay kit (Ambion, San Diego, CA) according to the directions of the assay kit manufacturer. Then, the integrity and content of extracted RNA was evaluated by using an Agilent 2100 BioAnalyzer (Agilent, San Jose, CA) according to the directions of the instrument manufacturer. In the next step, an NCode VILO cDNA synthesis assay kit (Qiagen, Germantown, MD) was used according to the directions of the assay kit manufacturer to create cDNA templates for real time quantitative PCR analysis, which was performed by using a QuantiFast SYBR Green master mix (Qiagen, Germantown, MD) according to the directions of the assay kit manufacturer to calculate the relative expression of circRNA_0079586, circRNA_RanGAP1, miR-183-5p, miR-877-3p, and MPO mRNA in each sample using the 2^−ΔΔCT^ approach. The real time quantitative PCR was carried out on a LightCycler 480 equipment (Roche, Basel, Switzerland) according to the directions of the equipment manufacturer.

### Cell culture and transfection

HUVEC cells were acquired from ATCC and cultured in a Dulbecco’s modified Eagle’s medium (DMEM, Gibco, Thermo Fisher Scientific, Waltham, MA) containing 4.5 g/l of glucose, 10% fetal bovine serum as well as streptomycin and streptomycin (Sigma Aldrich, St. Louis, MO). The culture was done in a 37° C humidified environment of 5% carbon dioxide and 95% air. When HUVEC cells were confluent, they were used to establish 5 cellular models as below. In cellular model I, HUVEC cells were divided into 5 groups, i.e., 1. WSS group (HUVEC cells subjected to WSS stress loading and treated with PBS); 2. TF group (HUVEC cells subjected to TF treatment); 3. TF + NC siRNA group (HUVEC cells subjected to TF treatment and then transfected with NC siRNA); 4. TF + circRNA_0079586 siRNA group (HUVEC cells subjected to TF treatment and then transfected with siRNA targeting circRNA_0079586); and 5. TF + circRNA_RanGAP1 siRNA group (HUVEC cells subjected to TF treatment and then transfected with siRNA targeting circRNA_RanGAP1). In cellular model II, HUVEC cells were divided into 4 groups, i.e., 1. NC group (HUVEC cells transfected with NC plasmid); 2. p-circRNA _0079586 group (HUVEC cells transfected with p-circRNA _0079586 plasmid); 3. p-circRNA_RanGAP1 group (HUVEC cells transfected with p-circRNA_RanGAP1 plasmid); and 4. p-circRNA_0079586 + p-circRNA_RanGAP1 group (HUVEC cells transfected with both p-circRNA_0079586 and p-circRNA_RanGAP1 plasmids). In cellular model III, HUVEC cells were divided into 4 groups, i.e., 1. NC siRNA group (HUVEC cells transfected with NC siRNA); 2. circRNA_0079586 siRNA group (HUVEC cells transfected with siRNA targeting circRNA_0079586); 3. circRNA_RanGAP1 siRNA group (HUVEC cells transfected with siRNA targeting circRNA_RanGAP1); and 4. circRNA_0079586 siRNA + circRNA_RanGAP1 siRNA group (HUVEC cells transfected with siRNAs targeting both circRNA_0079586 and circRNA_RanGAP1). In cellular model IV, HUVEC cells were divided into 2 groups, i.e., 1. NC group (HUVEC cells transfected with NC); 2. miR-183-5p mimics group (HUVEC cells transfected with miR-183-5p mimics). In cellular model V, HUVEC cells were divided into 2 groups, i.e., 1. NC group (HUVEC cells transfected with NC); 2. miR-887-3p mimics group (HUVEC cells transfected with miR-887-3p mimics). All transfection was done using the Lipofectamine RNAiMAX transfection reagent (Invitrogen, Carlsbad, CA) according to the directions of the transfection reagent manufacturer. All transfected cells were harvested 48 h later for subsequent analyses.

### Vector construction, mutagenesis and luciferase assay

Our sequence analysis showed that miR-183-5p could potentially target circRNA-0079586, so luciferase vectors containing wild type and mutant circRNA-0079586 were established and transfected into HUVEC cells with miR-183-5p to study the effect of miR-183-5p on circRNA-0079586 expression. Specifically, the prediction upon the interaction between circRNA-0079586 and miR-183-5p was accomplished by Circular RNA Interactome (https://circinteractome.nia.nih.gov/index.html). In brief, the wild type circRNA-0079586 sequence containing the miR-183-5p binding site was cloned into a pcDNA vector (Promega, Madison, WI) to generate a wild type circRNA-0079586 vector. Then, site directed mutagenesis was generated in the miR-183-5p binding site of circRNA-0079586, and the mutant type circRNA-0079586 sequence containing the mutated miR-183-5p binding site was also cloned into a pcDNA vector to generate a mutant type circRNA-0079586 vector. Then, both wild type and mutant type circRNA-0079586 vectors were co-transfected into HUVEC cells with miR-183-5p using the Lipofectamine RNAiMAX transfection reagent according to the directions of the transfection reagent manufacturer, and the luciferase activity of transfected cells was assayed 48 h later using a Dual Luciferase Assay kit (Promega, Madison, WI) according to the directions of the assay kit manufacturer. Similarly, our sequence analysis by Circular RNA Interactome showed that miR-877-3p could potentially target circRNA_RanGAP1, so luciferase vectors containing wild type and mutant circRNA_RanGAP1 were established and transfected into HUVEC cells with miR-877-3p to study the effect of miR-877-3p on circRNA_RanGAP1 expression using the methods described above. In addition, the prediction upon the interaction between miR-183-5p/miR-887-3p and MPO mRNA was accomplished by MirDB (www.mirdb.org). To study the effect of miR-877-3p and miR-183-5p on MPO expression, the wild type MPO promoter sequence containing the miR-183-5p or miR-877-3p binding site was cloned into pcDNA vectors, and site directed mutagenesis was generated in the miR-183-5p or miR-877-3p binding site of MPO, and the mutant type MPO sequence containing the mutated miR-183-5p or miR-877-3p binding site was also cloned into pcDNA vectors to generate mutant type MOP vectors. Then, both wild type and mutant type MPO vectors were co-transfected into HUVEC cells with miR-183-5p/miR-877-3p using the Lipofectamine RNAiMAX transfection reagent according to the directions of the transfection reagent manufacturer, and the luciferase activity of transfected cells was assayed 48 h later using a Dual Luciferase Assay kit.

### Western blot analysis

Total protein was isolated from each sample by using a RIPA buffer (Sigma Aldrich, St. Louis, MO) according to the directions of the buffer manufacturer. Then, after 15 min of centrifugation at 13,400 *g*, the protein supernatant was quantified by using a BCA protein assay (Sigma Aldrich, St. Louis, MO) according to the directions of the assay kit manufacturer to determine protein concentration. In the next step, 10 µg of total protein from each sample was separated on a 10% SDS-PAGE gel and blotted onto a PVDF membrane (Roche, Indianapolis, IN), which was then blocked at room temperature for 2 h in a TBST buffer containing 5% goat milk, and then probed in sequence with primary anti-MPO monoclonal antibody and HRP-conjugated IgG secondary antibody (Santa Cruz Biotechnology, Dallas, TX) according to the incubation conditions suggested by the antibody manufacturer. Finally, the target protein bands were identified by using an enhanced chemiluminescence reagent (ECL) (Sigma Aldrich, St. Louis, MO) according to the directions of the reagent manufacturer.

### Statistical analysis

All experiments were done ≥ 3 times. The results were ex-shown as mean ± SEM. Statistical analysis was done in SPSS 17.0 (SPSS, Chicago, IL) by utilizing the Student's t-tests for inter-group comparisons. *P* < 0.05 was deemed statistically significant.

## Results

### Clinical features of the patients

We recruited IA (intracranial aneurysm) patients and divided them into two groups based on the status of IA: RIAs (ruptured intracranial aneurysms) and UIAs (unruptured intracranial aneurysms). Tissue samples were collected after surgery from patients in both groups. The clinical features including sex, age, hypertension, smoking history, lesion size were compared between the two groups. No obvious difference was found between patients in the two groups (Table 1).

**Table 1:** Basic clinical features of recruited patients.

Characteristics	UIAs (N = 16)	RIAs (N = 18)	*P* value
Sex, male	10 (62.5)	14 (77.8)	0.713
Age, years	54.1 ± 5.9	53.5 ± 4.8	0.974
**Hypertension**			0.368
Yes	13 (81.3)	16 (83.3)	
No	3 (18.7)	2 (16.7)	
**Smoke history**			0.112
Yes	9 (56.3)	9 (50.0)	
No	7 (43.7)	9 (50.0)	
Size, cm	2.1 ± 0.4	1.9 ± 0.3	0.846
**Multiple**			0.259
Yes	3 (18.7)	5 (27.8)	
No	13 (81.3)	13 (72.2)	

### Shear stress reshaped the expression of circRNAs in the endothelial cells of IA patients

**Figure 1:**
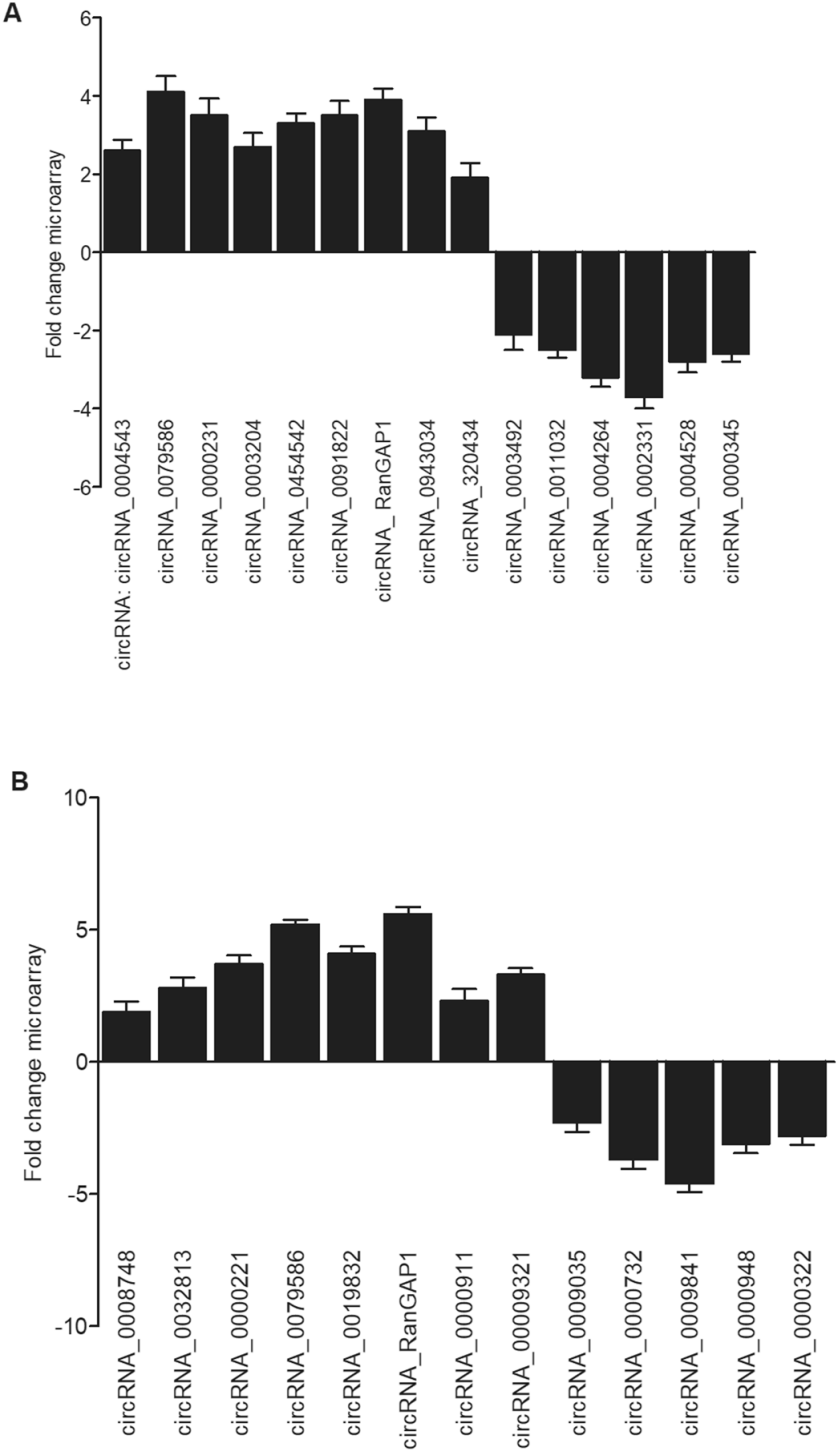
Different expression of circRNAs in UIAs and RIAs patients. (**A**) Microarray analysis showed up-regulated and down-regulated circRNAs in the endothelial cells collected from RIAs and UIAs patients. (**B**) Microarray analysis showed up-regulated and down-regulated circRNAs in the HUVECs treated with TF or WSS.

Previous reports revealed that circRNAs played crucial roles in the pathogenesis of IA. We collected the endothelial cells from IA tissue samples and subjected them to microarray screening to examine the differentially expressed circRNAs between RIAs and UIAs. The expression of circRNA_0004543, circRNA_0079586, circRNA_0000231, circRNA_0003204, circRNA_0454542, circRNA_0091822, circRNA_RanGAP1, circRNA_0943034 and circRNA_320434 was remarkably elevated in RIAs when compared with UIAs. On the contrary, the expression of circRNA_0003492, circRNA_0011032, circRNA_0004264, circRNA_0002331, circRNA_0004528 and circRNA_0000345 was notably suppressed in RIAs (Fig. 1A). Furthermore, we treated HUVECs with WSS (wall shear stress) and TF (turbulent flow), followed by circRNAs screening to find the differentially expressed circRNAs in HUVECs treated under the two conditions. The expression of circRNA_0008748, circRNA_0032813, circRNA_0000221, circRNA_0079586, circRNA_0019832, circRNA_RanGAP1, circRNA_0000911 and circRNA_00009321 was significantly enhanced in TF-treated HUVECs when compared with WSS-treated HUVECs, while the expression of circRNA_0009035, circRNA_0000732, circRNA_0009841, circRNA_0000948 and circRNA_0000322 was apparently repressed in TF-treated HUVECs when compared with WSS-treated HUVECs (Fig. 1B).

### Luciferase assays revealed the regulatory relationship between circRNA_0079586/miR-183-5p, circRNA_RanGAP1/miR-877-3p, miR-183-5p/MPO, and miR-877-3p/MPO

**Figure 2:**
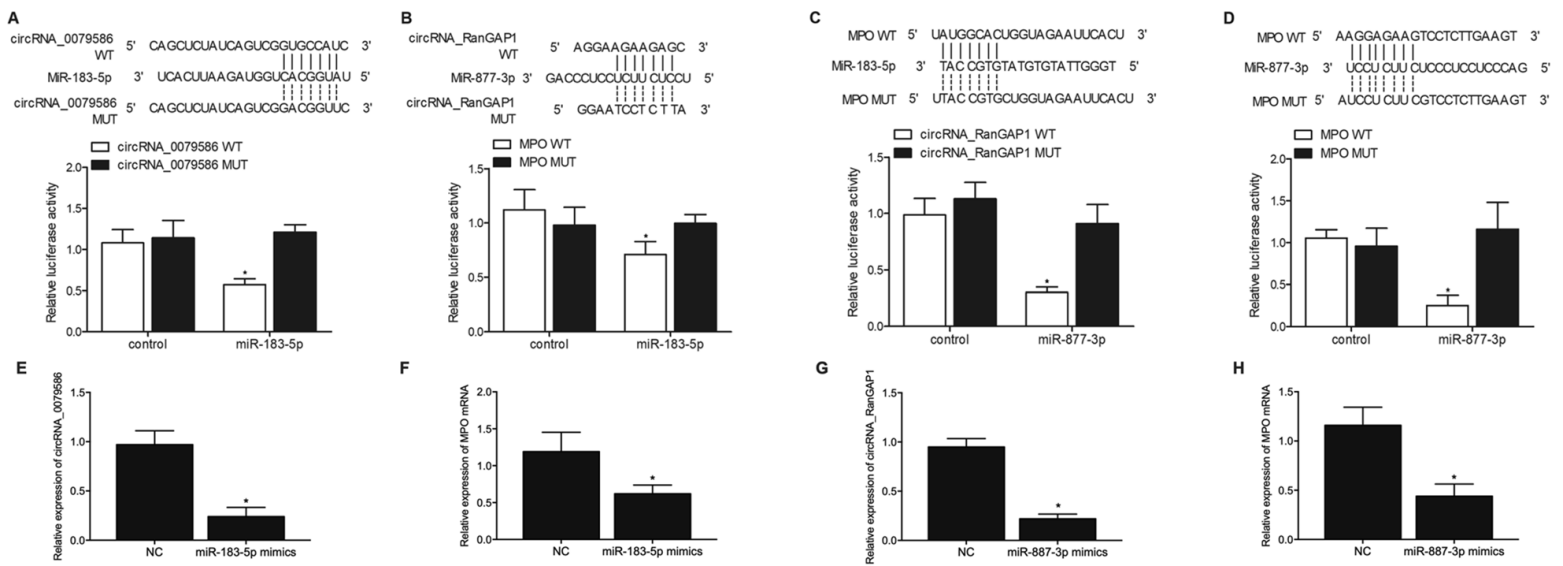
Luciferase assays revealed the regulatory relationships of circRNA_0079586/miR-183-5p, circRNA_RanGAP1/miR-877-3p, miR-183-5p/MPO, and miR-877-3p/MPO. (**A**) Sequence analysis and luciferase assay indicated that miR-183-5p could inhibit the expression of circRNA-0079586 (*P < 0.05 vs. circRNA_0079586 MUT). (**B**) Sequence analysis and luciferase assay indicated that miR-877-3p could inhibit the expression of circRNA-RanGAP1 (*P < 0.05 vs. MPO MUT). (**C**) Sequence analysis and luciferase assay indicated that miR-183-5p could inhibit the expression of MPO (*P < 0.05 vs. circRNA_RanGAP1 MUT). (**D**) Sequence analysis and luciferase assay indicated that miR-877-3p could inhibit the expression of MPO (*P < 0.05 vs. circRNA_MPO MUT). (**E**) Relative expression of circRNA-0079586 was inhibited by the overexpression of miR-183-5p (*P value < 0.05 vs. NC group). (**F**) Relative expression of MPO mRNA was inhibited by the overexpression of miR-183-5p (*P value < 0.05 vs. NC group). (**G**) Relative expression of circRNA-RanGAP1 was inhibited by the overexpression of miR-887-3p (*P value < 0.05 vs. NC group). (**H**) Relative expression of MPO mRNA was inhibited by the overexpression of miR-887-3p (*P value < 0.05 vs. NC group).

Sequence analysis showed that miR-183-5p could potentially target circRNA-0079586, so luciferase vectors containing wild type and mutant circRNA-0079586 were established and transfected into HUVEC cells with miR-183-5p. The luciferase activity of wild type circRNA-0079586 was significantly suppressed by miR-183-5p, while the luciferase activity of mutant circRNA-0079586 remained unchanged when compared with the control (Fig. 2A). Sequence analysis showed that miR-877-3p could potentially target circRNA-RanGAP1, so luciferase vectors containing wild type and mutant circRNA-RanGAP1 were established and transfected into HUVEC cells with miR-877-3p. The luciferase activity of wild type circRNA-RanGAP1 was significantly suppressed by miR-877-3p, while the luciferase activity of mutant circRNA-RanGAP1 remained unchanged when compared with the control (Fig. 2B). Sequence analysis showed that miR-183-5p could potentially target MPO, so luciferase vectors containing wild type and mutant MPO were established and transfected into HUVEC cells with miR-183-5p. The luciferase activity of wild type MPO was significantly suppressed by miR-183-5p, while the luciferase activity of mutant MPO remained unchanged when compared with the control (Fig. 2C). Sequence analysis showed that miR-877-3p could potentially target MPO, so luciferase vectors containing wild type and mutant MPO were established and transfected into HUVEC cells with miR-877-3p. The luciferase activity of wild type MPO was significantly suppressed by miR-877-3p, while the luciferase activity of mutant MPO remained unchanged when compared with the control (Fig. 2D). And to further verify the regulatory association between the miRNA and its possible target gene, we observed the relative expression of potential target genes in HUVEC cells overexpressing miR-183-5p or miR-887-3p. Accordingly, the relative expression of circRNA-0079586 (Fig. 2E) and MPO mRNA (Fig. 2F) were significantly down-regulated in HUVEC cells transfected with miR-183-45p mimics. And the relative expression of circRNA-RanGAP1 (Fig. 2G) and MPO mRNA (Fig. 2H) were down-regulated in cells transfected with miR-183-45p mimics.

### Different expression of circRNA-0079586, circRNA-RanGAP1, miR-183-5p, miR-877-3p and MPO mRNA in the endothelial cells collected from UIAs and RIAs patients

**Figure 3:**
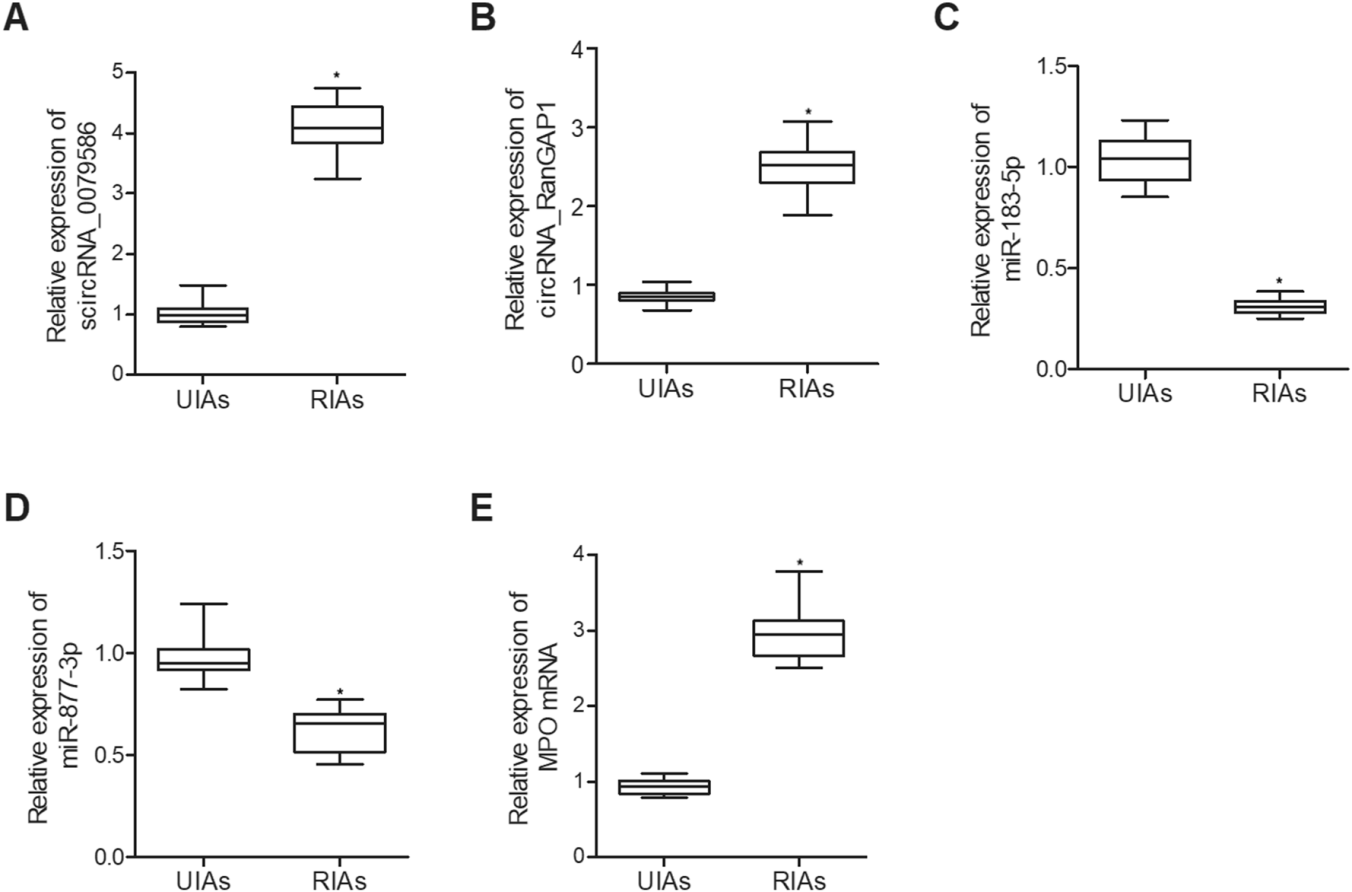
Different expression of circRNA-0079586, circRNA-RanGAP1, miR-183-5p, miR-877-3p and MPO in the endothelial cells collected from UIAs and RIAs patients. (**A**) The expression of circRNA-0079586 was enhanced in the endothelial cells collected from RIAs patients (*P < 0.05 vs. UIAs). (**B**) The expression of circRNA-RanGAP1 was enhanced in the endothelial cells collected from RIAs patients (*P < 0.05 vs. UIAs). (**C**) The expression of miR-183-5p was inhibited in the endothelial cells collected from RIAs patients (*P < 0.05 vs. UIAs). (**D**) The expression of miR-877-3p was inhibited in the endothelial cells collected from RIAs patients (*P < 0.05 vs. UIAs). (**E**) The expression of MPO mRNA was enhanced in the endothelial cells collected from RIAs patients (*P < 0.05 vs. UIAs).

Quantitative real time PCR was performed to analyze the expression of circRNA-0079586, circRNA-RanGAP1, miR-183-5p, miR-877-3p and MPO mRNA in the endothelial cells collected from UIAs and RIAs patients. The expression of circRNA-0079586 (Fig. 3A) and circRNA-RanGAP1 (Fig. 3B) was remarkably increased in endothelial cells from RIAs patients. The expression of miR-183-5p (Fig. 3C) and miR-877-3p (Fig. 3D) was notably decreased in endothelial cells from RIAs patients. The expression of MPO mRNA (Fig. 3E) was apparently increased in endothelial cells from RIAs patients.

### Alteration of circRNA-0079586 and circRNA-RanGAP1 expression significantly changed the expression of miR-183-5p, miR-877-3p and MPO in TF-treated HUVECs

**Figure 4:**
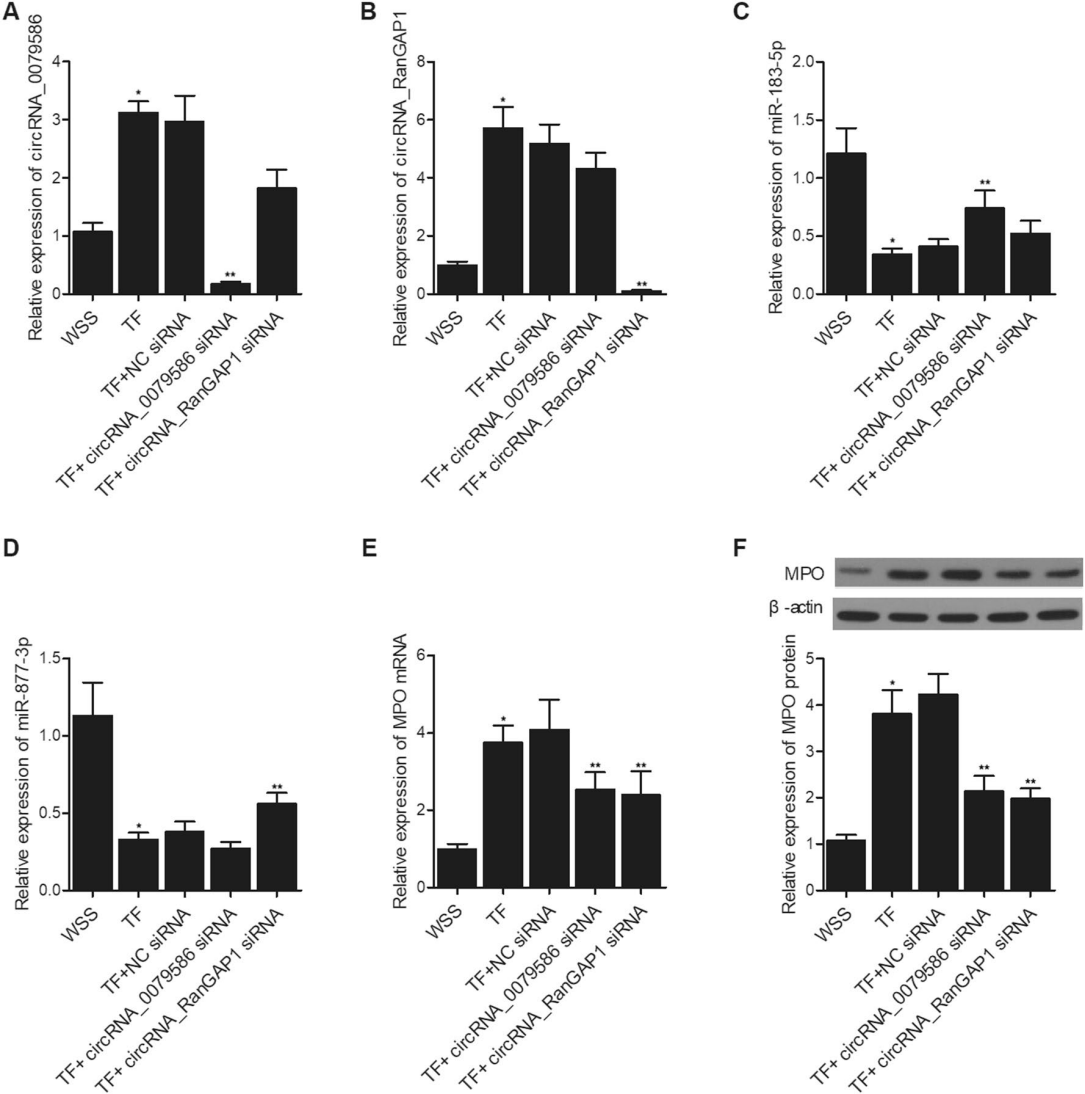
Down-regulation of circRNA_0079586 and circRNA-RanGAP1 with siRNA altered the expression of circRNA-0079586, circRNA-RanGAP1, miR-183-5p, miR-877-3p and MPO in WSS- and TF-treated HUVECs. (**A**) CircRNA-0079586 siRNA dramatically suppressed the expression of circRNA-0079586 in TF-treated HUVECs (*P < 0.05 vs. WSS; **P < 0.05 vs. TF + NC siRNA). (**B**) CircRNA-RanGAP1 siRNA dramatically suppressed the expression of circRNA-RanGAP1 in TF-treated HUVECs (*P < 0.05 vs. WSS; **P < 0.05 vs. TF + NC siRNA). (**C**) CircRNA-0079586 siRNA significantly enhanced the expression of miR-183-5p in TF-treated HUVECs (*P < 0.05 vs. WSS; **P < 0.05 vs. TF + NC siRNA). (**D**) CircRNA-RanGAP1 siRNA significantly enhanced the expression of miR-877-3p in TF-treated HUVECs (*P < 0.05 vs. WSS; **P < 0.05 vs. TF + NC siRNA). (**E**) CircRNA-0079586 siRNA and circRNA-RanGAP1 siRNA decreased the expression of MPO mRNA in TF-treated HUVECs (*P < 0.05 vs. WSS; **P < 0.05 vs. TF + NC siRNA). (**F**) CircRNA-0079586 siRNA and circRNA-RanGAP1 siRNA decreased the expression of MPO protein in TF-treated HUVECs (*P < 0.05 vs. WSS; **P < 0.05 vs. TF + NC siRNA).

CircRNA-0079586 and circRNA-RanGAP1 siRNAs were transfected into TF-treated HUVECs to evaluate its effect on the expression of circRNA-0079586, circRNA-RanGAP1, miR-183-5p, miR-877-3p and MPO. The expression of circRNA-0079586 was dramatically suppressed in TF-treated HUVECs by circRNA-0079586 siRNA (Fig. 4A). The expression of circRNA-RanGAP1 was dramatically suppressed in TF-treated HUVECs by circRNA-RanGAP1 siRNA (Fig. 4B). TF treatment remarkably down-regulated the expression of miR-183-5p and miR-877-5p when compared with WSS treatment. The expression of miR-183-5p was significantly activated in TF-treated HUVECs by circRNA-0079586 siRNA (Fig. 4C). The expression of miR-877-3p was significantly activated in TF-treated HUVECs by circRNA-RanGAP1 siRNA (Fig. 4D). The expression of MPO mRNA and protein was notably up-regulated in TF-treated HUVECs when compared with WSS-treated HUVECs. CircRNA-0079586 and circRNA-RanGAP1 siRNAs obviously decreased the expression of MPO mRNA (Fig. 4E) and protein (Fig. 4F) in TF-treated HUVECs.

**Figure 5:**
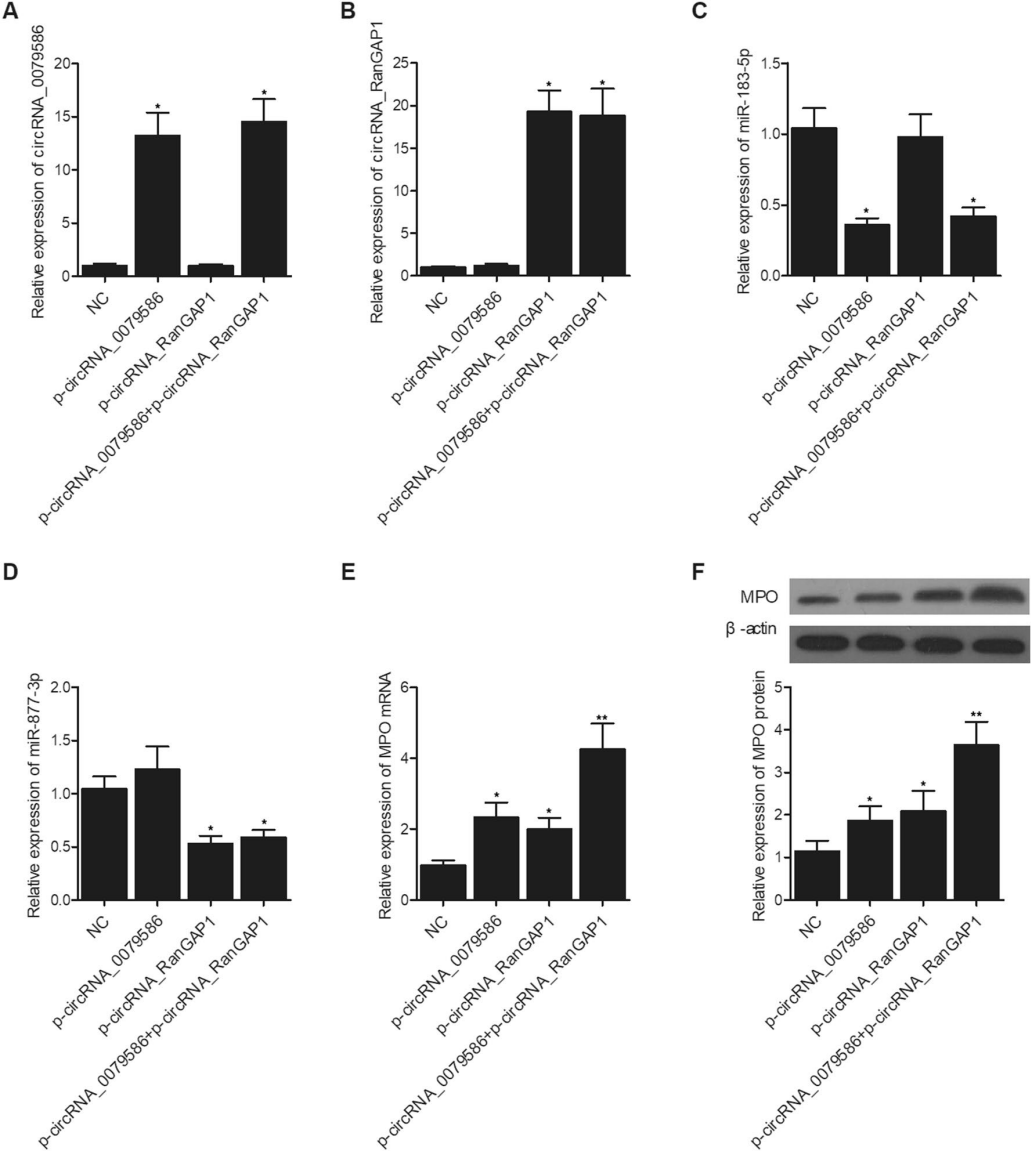
Overexpression of circRNA_0079586 and circRNA-RanGAP1 suppressed the expression of miR-183-5p and miR-877-3p, respectively, and activated the expression of MPO in HUVECs. (**A**) The expression of circRNA-0079586 was dramatically enhanced in HUVECs transfected with p-circRNA-0079586 and p-circRNA_0079586 + p-circRNA_RanGAP1 (*P < 0.05 vs. NC). (**B**) The expression of circRNA-RanGAP1 was dramatically enhanced in HUVECs transfected with p-circRNA-RanGAP1 and p-circRNA_0079586 + p-circRNA_RanGAP1 (*P < 0.05 vs. NC). (**C**) The expression of miR-183-5p was obviously suppressed in HUVECs transfected with p-circRNA-0079586 and p-circRNA_0079586 + p-circRNA_RanGAP1 (*P < 0.05 vs. NC). (**D**) The expression of miR-877-3p was apparently suppressed in HUVECs transfected with p-circRNA-RanGAP1 and p-circRNA_0079586 + p-circRNA_RanGAP1 (*P < 0.05 vs. NC). (**E**) The expression of MPO mRNA was significantly increased in HUVECs transfected with p-circRNA-0079586 or p-circRNA_RanGAP1 alone, and to a higher extent in HUVECs transfected with p-circRNA_0079586 + p-circRNA_RanGAP1 (*P < 0.05 vs. NC; **P < 0.05 vs. p-circRNA_0079586). (**F**) The expression of MPO protein was significantly increased in HUVECs transfected with p-circRNA-0079586 or p-circRNA_RanGAP1 alone, and to a higher extent in HUVECs transfected with p-circRNA_0079586 + p-circRNA_RanGAP1 (*P < 0.05 vs. NC; **P < 0.05 vs. p-circRNA_0079586).

**Figure 6:**
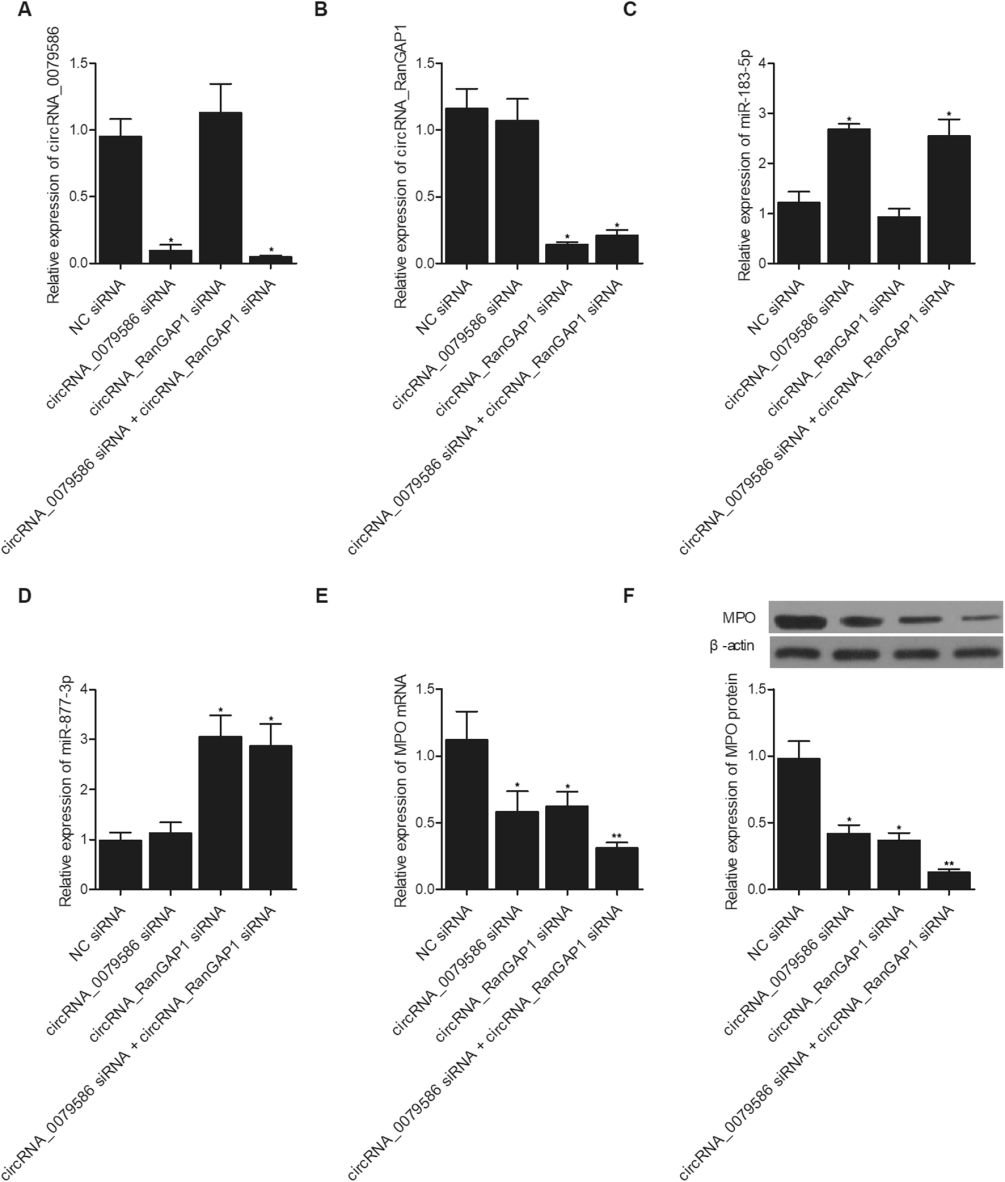
Suppression of circRNA_0079586 and circRNA-RanGAP1 enhanced the expression of miR-183-5p and miR-877-3p, respectively, and repressed the expression of MPO in HUVECs. (**A**) The expression of circRNA-0079586 was dramatically suppressed in HUVECs transfected with circRNA_0079586 siRNA and circRNA_0079586 siRNA + circRNA_RanGAP1 siRNA (*P < 0.05 vs. NC siRNA). (**B**) The expression of circRNA-RanGAP1 was dramatically repressed in HUVECs transfected with circRNA_RanGAP1 siRNA and circRNA_0079586 siRNA + circRNA_RanGAP1 siRNA (*P < 0.05 vs. NC siRNA). (**C**) The expression of miR-183-5p was obviously activated in HUVECs transfected with circRNA_0079586 siRNA and circRNA_0079586 siRNA + circRNA_RanGAP1 siRNA (*P < 0.05 vs. NC siRNA). (**D**) The expression of miR-877-3p was apparently enhanced in HUVECs transfected with circRNA_RanGAP1 siRNA and circRNA_0079586 siRNA + circRNA_RanGAP1 siRNA (*P < 0.05 vs. NC siRNA). (**E**) The expression of MPO mRNA was significantly inhibited in HUVECs transfected with circRNA_0079586 siRNA or circRNA_RanGAP1 siRNA alone, and to a higher extent in HUVECs transfected with circRNA_0079586 siRNA + circRNA_RanGAP1 siRNA (*P < 0.05 vs. NC siRNA; **P < 0.05 vs. circRNA_0079586 siRNA). (**F**) The expression of MPO protein was significantly inhibited in HUVECs transfected with circRNA_0079586 siRNA or circRNA_RanGAP1 siRNA alone, and to a higher extent in HUVECs transfected with circRNA_0079586 siRNA + circRNA_RanGAP1 siRNA (*P < 0.05 vs. NC siRNA; **P < 0.05 vs. circRNA_0079586 siRNA).

In order to further explore the effect of circRNA-0079586 and circRNA-RanGAP1 expression on the expression of miR-183-5p, miR-877-3p and MPO, we overexpressed and repressed the expression of circRNA-0079586 and circRNA-RanGAP1 in HUVECs. The expression of circRNA-0079586 was dramatically enhanced in HUVECs transfected with p-circRNA-0079586 and p-circRNA_0079586 + p-circRNA_RanGAP1 (Fig. 5A). The expression of circRNA- RanGAP1 was dramatically enhanced in HUVECs transfected with p-circRNA-RanGAP1 and p-circRNA_0079586 + p-circRNA_RanGAP1 (Fig. 5B). The expression of miR-183-5p was obviously suppressed in HUVECs transfected with p-circRNA-0079586 and p-circRNA_0079586 + p-circRNA_RanGAP1 (Fig. 5C). The expression of miR-877-3p was apparently suppressed in HUVECs transfected with p-circRNA-RanGAP1 and p-circRNA_0079586 + p-circRNA_RanGAP1 (Fig. 5D). The expression of MPO mRNA (Fig. 5E) and protein (Fig. 5F) was significantly increased in HUVECs transfected with p-circRNA-0079586 or p-circRNA_RanGAP1 alone, and to a higher extent in HUVECs transfected with p-circRNA_0079586 + p-circRNA_RanGAP1. On the contrary, the expression of circRNA-0079586 was dramatically suppressed in HUVECs transfected with circRNA_0079586 siRNA and circRNA_0079586 siRNA + circRNA_RanGAP1 siRNA (Fig. 6A). The expression of circRNA-RanGAP1 was dramatically repressed in HUVECs transfected with circRNA_RanGAP1 siRNA and circRNA_0079586 siRNA + circRNA_RanGAP1 siRNA (Fig. 6B). The expression of miR-183-5p was obviously activated in HUVECs transfected with circRNA_0079586 siRNA and circRNA_0079586 siRNA + circRNA_RanGAP1 siRNA (Fig. 6C). The expression of miR-877-3p was apparently enhanced in HUVECs transfected with circRNA_RanGAP1 siRNA and circRNA_0079586 siRNA + circRNA_RanGAP1 siRNA (Fig. 6D). The expression of MPO mRNA (Fig. 6E) and protein (Fig. 6F) was significantly inhibited in HUVECs transfected with circRNA_0079586 siRNA or circRNA_RanGAP1 siRNA alone, and to a higher extent in HUVECs transfected with circRNA_0079586 siRNA + circRNA_RanGAP1 siRNA.

## Discussion

In this study, we recruited UIAs and RIAs patients and collected their tissue samples to compare the different expression of circRNAs using microarray screening. We found a batch of up-regulated and down-regulated circRNAs between RIAs and UIAs patients. In addition, we performed luciferase assay to explore the regulatory relationship between circRNAs and miRNAs, as well as between the miRNAs and their target genes. The luciferase activity of circRNA-0079586 was suppressed by miR-183-5p and the luciferase activity of circRNA-RanGAP1 was suppressed by miR-877-3p. The luciferase activity of MPO was inhibited by miR-183-5p and miR-877-3p.

High WSS values may be detected at possible locations of IA rupture as well as the neck section of IA lesions^34,35^. Also, experimental IA models with IA rupture induced by high WSS gradients showed that IA is extremely sensitive to the diseases of intracranial arteries induced by high WSS^36,37^. The effect of WSS on IA rupture remains unclear, although WSS with excessively reduced focal hemodynamics may lead to structural fragility as well as reduced resistibility of the aneurysmal wall^38^. A reduced magnitude of WSS can also enhance chronic inflammation as well as atherosclerotic changes induced by macrophages, which in turn cause the thinning of the aneurysmal wall to cause further rupture^39^. In this study, we performed qPCR to compare the different expression of circRNA-0079586, circRNA-RanGAP1, miR-183-5p, miR-877-3p and MPO in the endothelial cells collected from UIAs and RIAs patients. The expression of circRNA-0079586 and circRNA-RanGAP1 was significantly enhanced in RIAs patients when compared with UIAs patients. The expression of miR-183-5p and miR-877-3p was remarkably repressed in RIAs patients when compared with UIAs patients. The expression of MPO was significantly activated in RIAs patients when compared with UIAs patients.

Wang et al. used high throughput sequencing to screen the effects of circRNAs on GM and paired non-cancer tissues. They found that circ_0079586 was up-regulated in GM specimens^40^. Located at chr7: 23353140—23383472 and containing 586 base pairs, circ_0079586 is generated by the splicing of IGF2BP3. In this study, we altered the expression of circRNA-0079586 and circRNA-RanGAP1 in HUVECs. The expression of circRNA-0079586 and circRNA-RanGAP1 was negatively correlated with the expression of miR-183-5p and miR-877-3p, respectively, but positively correlated with the expression of MPO mRNA and protein. Located on chromosome 7, miR-183 has been shown to play a role as prospective oncogene in lung cancer, prostate cancer, breast cancer, colorectal cancer as well as hepatocellular cancer^41^. In general, miR-183-5p is either abnormally upregulated or downregulated in different types of cancers^42^. As an example, miR-183-5p is upregulated in hepatocellular cancer to inhibit cancer cell apoptosis by suppressing PDCD4 expression^43^. However, miR-183-5p also inhibited the invasion of gastric cancer by targeting Ezrin^44^. In one study, circ-RanGAP1 expression was found to be considerably upregulated in GC tissues, especially in the later stage of GC^45^. MiR-877-3p was shown to promote the differentiation and proliferation of lung and bladder cancer cells^46,47^. Also, miR-877-3p was shown to inhibit the differentiation of mesenchymal stem cells in the lungs to myofibroblasts to alleviate lung fibrosis caused by bleomycin^46^. It was also suggested that circ-RanGAP1 can promote the invasion and metastasis of GC by sponging the expression of miR-877-3p, a tumor suppressor^40^. Another research signified that miR-877 might serve as a tumor suppressor to block the expansion of hepatocellular carcinoma cells^48^.

In the presence of H2O2, MPO is generated by neutrophilic respiratory burst to promote the production of chlorinating agents such as hypochlorous acid. Besides its prominent role in protection against microbes, MPO has also been implicated in the destabilization of atherosclerotic plaques^49^. It was actually found that a short treatment using a low dose of Ox-LDL noticeably boosted the number EC-binding monocytes as well as the expression of ICAM-1 on the surface of EC cells. Furthermore, a prolonged treatment using a high dose of Ox-LDL caused increased cytotoxicity as well as EC monolayer detachment, indicating that Ox-LDL stimulation may lead to atherosclerosis by promoting the expression of adhesion molecules on EC surface. In cerebral aneurysm patients, the concentrations of circulating MPO are locally increased in CA sacs to enhance the number of MPO-positive cells such as neutrophils in the aneurysm. In a CA mouse model, the deficiency of MPO reduced the levels of pro-inflammatory molecules in cerebral arteries while lowering the number of leukocytes to alleviate the rupture of cerebral aneurysm^50^. Since MPO shortage is commonly seen in human populations, it is important to analyze the rate of cerebral aneurysm in MPO deficiency^31^. In UIA patients, MPO showed the association with the danger of aneurysm rupture, indicating that MPO may be used as a potential biomarker for aneurysm rupture^51^.

## Conclusion

In this study, we established two MPO-modulating signaling pathways of circRNA_0079586/miR-183-5p/MPO and circRNA_RanGAP1/ miR-877-3p/MPO. These two signaling pathways are involved in the pathogenesis of intracranial aneurysms rupture. In patients with ruptured intracranial aneurysms, the expression of MPO was up-regulated with promoted expression of circRNA_0079586 and circRNA_RanGAP1.

## Supplementary Information

Supplementary Information 1.

## Data Availability

The datasets used and/or analysed during the current study are available from the corresponding author on reasonable request.

## Abbreviations

HUVECs: Human umbilical endothelial cells
IA: Intracranial aneurysm
RIAs: Ruptured intracranial aneurysms
UIAs: Unruptured intracranial aneurysms
WSS: Wall shear stress
TF: Turbulent flow
