# Supplementary material for: CircRNA_0079586 and circRNA_RanGAP1 are involved in the pathogenesis of intracranial aneurysms rupture by regulating the expression of MPO
Source: Sci Rep. 2021 Oct 5;11:19800. doi: 10.1038/s41598-021-99062-w (PMC8492745; doi:10.1038/s41598-021-99062-w)
Supplement: Supplementary file 1 — Supplementary Information 1. [file 41598_2021_99062_MOESM1_ESM.pdf]

CircRNA_0079586 and circRNA_RanGAP1 are involved in the pathogenesis of intracranial aneurysms rupture by regulating the expression of MPO

Zhuang Zhang[1], Rubo Sui[1], Lili Ge[2], Dongjian Xia[3*]

1. Department of Neurology, the First Affiliated Hospital, Jinzhou Medical University, Jinzhou, China

2. Department of Ultrasound, the First Affiliated Hospital, Jinzhou Medical University, Jinzhou, China

3. Department of Neurosurgery, the First Affiliated Hospital, Jinzhou Medical University, Jinzhou, China

[*] Correspondence to: Dongjian Xia

Affiliation: Department of Neurosurgery, the First Affiliated Hospital, Jinzhou Medical University, Jinzhou, China

Address: Renmin Street, No.2, Fifth Duan, Jinzhou, China.

Email: conditionnerve@163.com

Original WB cuts

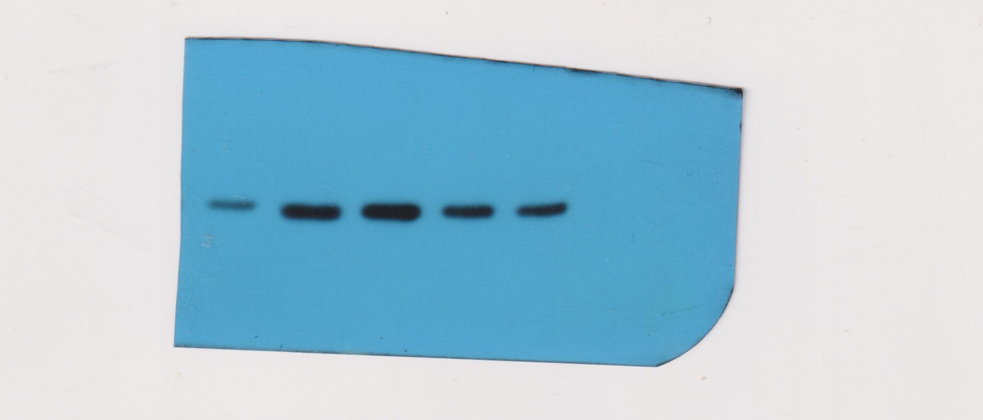

Fig 4F-MPO

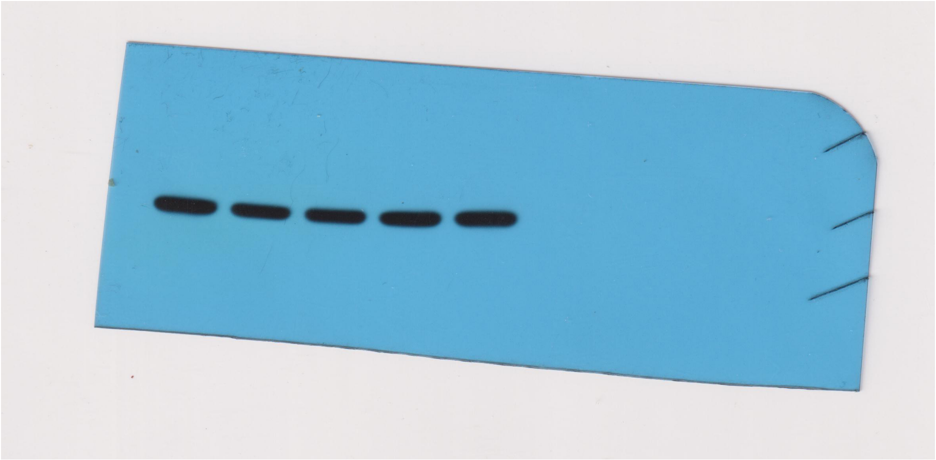

Fig 4F-beta actin

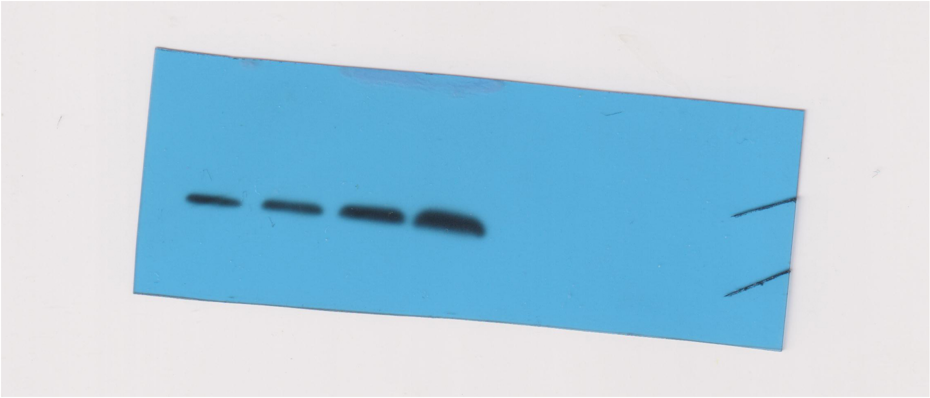

Fig 5F-MPO

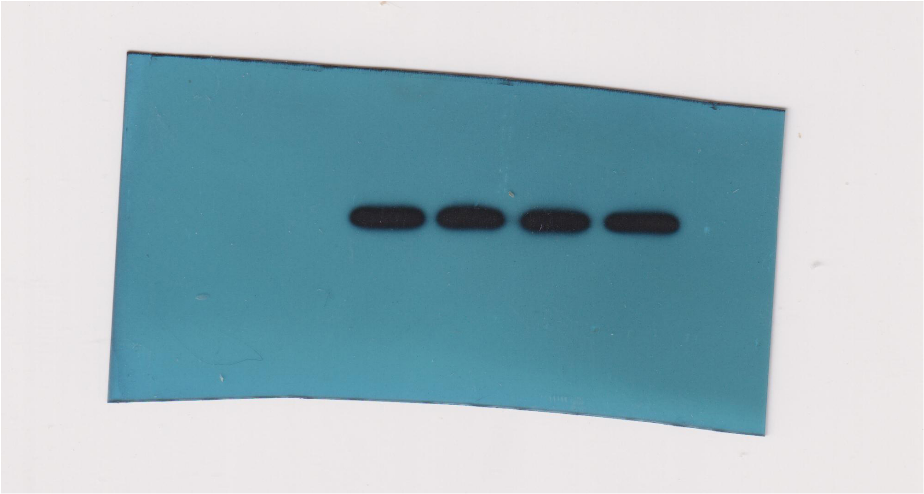

Fig 5F-beta actin

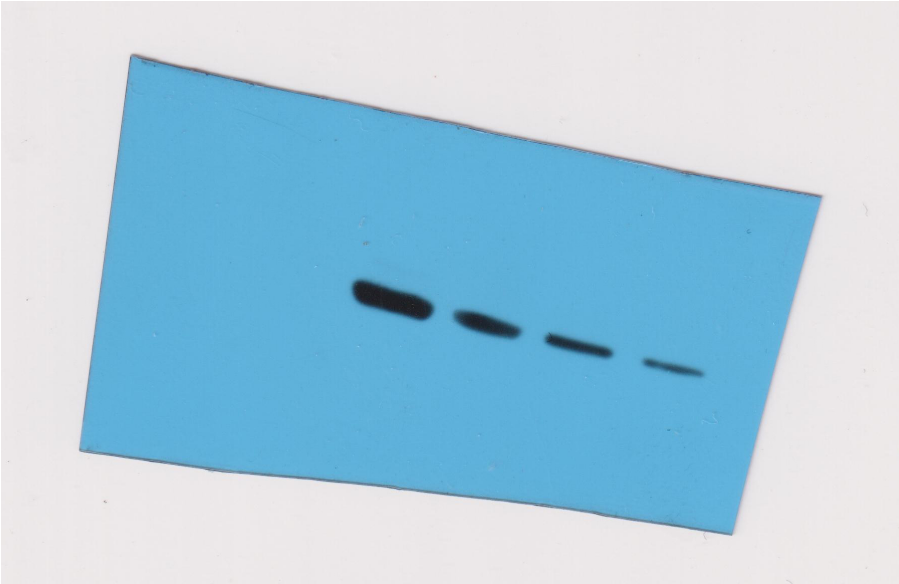

Fig 6F-MPO

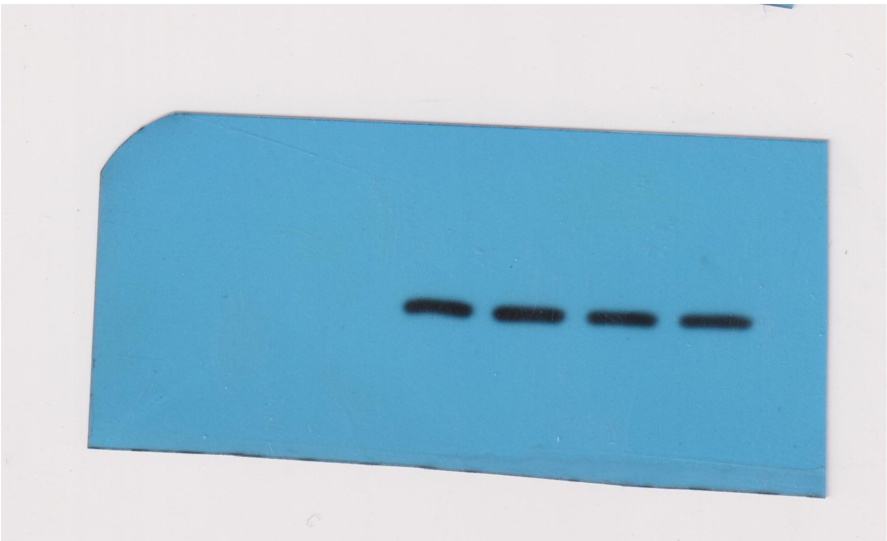

Fig 6F-beta actin
